# Development of one-step TaqMan^®^ real-time reverse transcription-PCR and conventional reverse transcription-PCR assays for the detection of equine rhinitis A and B viruses

**DOI:** 10.1186/1746-6148-8-120

**Published:** 2012-07-25

**Authors:** Zhengchun Lu, Peter J Timoney, Jena White, Udeni BR Balasuriya

**Affiliations:** 1 Maxwell H. Gluck Equine Research Center, Department of Veterinary Science, University of Kentucky, 108 Maxwell H, Lexington, KY, 40546, USA

**Keywords:** Real-time RT-PCR, RT-PCR, Equine rhinitis virus A, Equine rhinitis virus B

## Abstract

**Background:**

Equine rhinitis viruses A and B (ERAV and ERBV) are common equine respiratory viruses belonging to the family *Picornaviridae*. Sero-surveillance studies have shown that these two viral infections are prevalent in many countries. Currently, the diagnosis of ERAV and ERBV infections in horses is mainly based on virus isolation (VI). However, the sensitivity of VI testing varies between laboratories due to inefficient viral growth in cell culture and lack of cytopathic effect. Therefore, the objective of this study was to develop molecular diagnostic assays (real-time RT-PCR [rRT-PCR] and conventional RT-PCR [cRT-PCR] assays) to detect and distinguish ERAV from ERBV without the inherent problems traditionally associated with laboratory diagnosis of these infections.

**Results:**

Three rRT-PCR assays targeting the 5'-UTR of ERAV and ERBV were developed. One assay was specific for ERAV, with the two remaining assays specific for ERBV. Additionally, six cRT-PCR assays targeting the 5'-UTR and 3D polymerase regions of ERAV and ERBV were developed. Both rRT-PCR and cRT-PCR assays were evaluated using RNA extracted from 21 archived tissue culture fluid (TCF) samples previously confirmed to be positive for ERAV (n = 11) or ERBV (n = 10) with mono-specific rabbit antisera. The ERAV rRT-PCR and cRT-PCR assays could only detect ERAV isolates and not ERBV isolates. Similarly, the ERBV rRT-PCR and cRT-PCR assays could only detect ERBV isolates and not ERAV isolates. None of the rRT-PCR or cRT-PCR assays cross-reacted with any of the other common equine respiratory viruses. With the exception of one cRT-PCR assay, the detection limit of all of these assays was 1 plaque forming unit per ml (pfu/ml).

**Conclusion:**

The newly developed rRT-PCR and cRT-PCR assays provide improved diagnostic capability for the detection and differentiation of ERAV and ERBV. However, a larger number of clinical specimens will need to be tested before each assay is adequately validated for the detection of ERAV and/or ERBV in suspect cases of either viral infection.

## Background

The family *Picornaviridae* is a large family of viruses classified into several genera with extensive diversity in physical properties, antigenicity and mechanisms of pathogenesis [1]. Although there are many different picornaviruses with various degrees of relatedness, all share several features in common. The picornaviruses have a single-stranded positive-sense RNA genome with a 5'-end covalently linked to a VPg (virion protein genome-linked) protein. The RNA genome contains a 5′ untranslated region (UTR) with an internal ribosome entry site (IRES), a single open reading frame (ORF) encoding the viral capsid proteins and the viral replicase proteins, a 3′ UTR and a 3′ poly(A) tail [2]. The ORF is divided into three regions: P1 encodes four structural proteins (VP1-VP4); P2 (2A, 2B, 2C and 2B3) and P3 (3A, 3B, 3B2, 3C and 3D) encode nine non-structural proteins [3]. A key component of the replication machinery is the RNA-dependent RNA polymerase (RdRp), also referred to as 3D polymerase (3D^pol^) in picornaviruses. This protein is responsible for the synthesis of both plus- and minus-strand viral RNA [4]. Equine picornaviruses, formerly known as equine rhinoviruses 1 and 2, have been reclassified as equine rhinitis A virus (ERAV) and equine rhinitis B virus (ERBV). ERAV (formerly equine rhinovirus 1 [prototype ERAV.P393/76]), a member of the genus *Aphthovirus* in the family *Picornaviridae*, was first isolated in the United Kingdom in 1962 [5-7]. The genome organization and structure of ERAV is very similar to that of other Picornaviruses (e.g. foot-and-mouth disease virus). The second equine rhinitis virus, ERBV (formerly equine rhinovirus 2 [prototype P1436/71]) was first isolated in Switzerland and subsequent sequence determination resulted in it being classified in a new genus *Erbovirus*, also in the family *Picornaviridae*[7,8]. There are three ERBV serotypes (designated ERBV 1, 2 and 3) that are differentiated on the basis of their acid lability/stability, genetic sequences and neutralization by type-specific antisera. The ERBV1 and ERBV3 serotypes comprise two distinct phylogenetic groups, one of which is phenotypically acid labile (ERBV1; [9]) and the other is acid stable (ERBV3; [10]). Subsequently, a third equine rhinovirus virus (equine rhinovirus 3) was also isolated in Switzerland and following sequence analysis of its viral capsid proteins, it was shown to be a second serotype in the genus *Erbovirus*, and was designated as ERBV2 (prototype P313/75) [9,11,12].

Strains of ERAV, ERBV1 and ERBV2 have been identified from both subclinical and clinical upper respiratory tract infections in horses worldwide [13-16]. Little is known about the pathogenesis of ERAV and ERBV, which could be attributable in part to the lack of suitable laboratory methods for the diagnosis of these infectious agents. Seroprevalence data reported by different investigators indicate that neutralizing antibodies to ERAV and ERBV can be found in 50% to 80% of horses worldwide and the seropositive percentage seems to be correlated with the age of the animals [15,17-20]. Most ERAV, ERBV1 and ERBV2 isolates were recovered from horses with acute febrile respiratory disease with clinical signs of high fever for 1–3 days, serous to mucopurulent nasal discharge, anorexia, leg edema and enlarged lymph nodes of the head and neck that were sensitive on palpation. A significant number of horses may carry and shed virus in their urine for an extended period of time [16]. Subclinical infection and subsequent seroconversion have also been reported [5,16,21,22].

The clinical signs of equine influenza virus (EIV), equine herpesvirus-1 and -4 (EHV-1 and EHV-4), equine adenovirus 1 (EAdV1), equine arteritis virus (EAV), and equine rhinitis A and B (ERAV, ERBV1, ERBV2) infections are very similar and resemble a number of other infectious and non-infectious equine respiratory diseases [23,24]. Accordingly, a provisional clinical diagnosis based solely on the respiratory signs must be confirmed by laboratory testing. Furthermore, rapid and accurate identification of these viruses is critical for the control of the diseases they cause. Therefore, the development of rapid, highly sensitive and specific diagnostic assays is essential for the identification and differentiation of ERAV and ERBV in infected horses during outbreaks of disease. In addition, such assays would facilitate epidemiological investigations.

Traditionally, ERAV and ERBV have been detected by virus isolation (VI) in susceptible cells lines such as African green monkey kidney (Vero) or rabbit kidney-13 (RK-13) cells. Sources of these viruses can include nasal swabs, blood, feces and urine [16,25,26]. VI can be challenging because some strains of these viruses may grow poorly in cell culture and may not give rise to visible cytopathic effect [27,28]. A modified culture medium supplemented with MgCl_2_ can enhance the growth of some ERBV strains, but it is unsuitable for diagnostic purposes due to lack of sensitivity [27]. Furthermore, successful VI frequently requires multiple blind passages and subsequent confirmation by electron microscopy or immunofluorescence testing in the case of non-cytopathic strains. ERAV and ERBV infection can also be detected serologically by demonstration of a four-fold or greater rise in antibody titer between acute and convalescent (paired) sera by virus neutralization [8,16,29] or complement fixation tests [13,30], however, serology might not be helpful in acute outbreak situations due to the time delay of the convalescent result. Furthermore, these traditional serologic techniques, although sensitive and specific, are time consuming and tedious. Several rapid molecular tests such as conventional RT-PCR (cRT-PCR) and real-time RT-PCR (rRT-PCR) have been developed for ERAV and ERBV [27,31-33]. The primers used in these assays were located in the 3D^pol^, 3'-UTR, 5'-UTR or VP1-2A regions of the viral genome. In the present study, we developed a panel of three new rRT-PCR assays for ERAV and ERBV targeting the 5'-UTR region of each viral genome, respectively. In addition, to facilitate more diagnostic flexibility, a panel of six cRT-PCR assays targeting the 5'-UTR and 3D^pol^ regions of ERAV or ERBV was also developed. This would allow diagnostic laboratories that do not have functional real-time RT-PCR assays to diagnose ERAV and ERBV infections in horses.

## Results and discussion

### Development of rRT-PCR assays for the detection of ERAV and ERBV

Three ERAV and ERBV specific primer and probe sets were developed targeting the conserved 5'-UTR region of the viral genomes (Table 1). One primer and probe set was specific for ERAV (named ERAV rRT-PCR assay) and the primers were degenerated to accommodate nucleotide variations found in sequences that are available in GenBank (n = 8). The second primer and probe set (named ERBV1 rRT-PCR assay) was specific for ERBV1 strain. A third assay (named ERBV2 rRT-PCR assay) consisted of the same reverse primer and probe sequences as in the ERBV1 assay with the exception that the forward primer was specific for ERBV2. The 4-nucleotide difference in the forward primer between the ERBV1 and ERBV2 assays was designed to increase the likelihood of detection of ERBV2 strains. Three rRT-PCR assays were initially tested with prototype strains of ERAV and ERBV obtained from the USDA’s National Veterinary Services Laboratories (NVSL), Ames, IA. All the assays were optimized using RNA extracted from the prototype strains of ERAV and ERBV and with different primers and probe concentrations using TaqMan^®^ one-step RT-PCR master mix in a checkerboard assay. The optimal primer and probe concentrations producing the greatest sensitivity and specificity for detection of ERAV and ERBV were selected for the final assay as described in the materials and methods section. The ERAV assay detected only the ERAV prototype strain with a mean cycle threshold (Ct) of 21.79 ± 0.30 (ranging from 21.58 to 22.00) and no cross-reaction with the ERBV prototype strain was noted. Both ERBV rRT-PCR assays detected only the ERBV prototype strain, but not the ERAV strain, with a mean Ct value of 12.99 ± 0.01 (ranging from 12.98 to 13) for the ERBV1 rRT-PCR assay and 29.01 ± 0.02 (ranging from 29.00 to 29.03) for the ERBV2 rRT-PCR assay, respectively. Subsequently, the specificity of these assays was tested using a range of other equine respiratory viruses including EAV, EIV, EAdV1 and 2, EHV 1-5 and Salem virus. The assays were shown to be 100% specific with no cross-reactivity with nucleic acid extracted from the afore-mentioned equine respiratory pathogens.

**Table 1:** Primers and probes used in the rRT-PCR assays

**rRT-PCR Assay Name**	**Target Genes (GenBank Accession Number)**	**Primer or Probe Names**^a^	**Sequence 5' to 3' and Nucleotide Location**	**Length of Fragment (bp)**
ERAV	5'-UTR of ERAV (L43052)	ERAV F	AGCGGCK^**d**^TGCTGGATTTTC (397-415)	60
		ERAV R	CATY^**e**^TGYCAGCTTGGTGACA (438-457)	
		ERAV Pr	FAM^b^-CGGTGCCATTGCT-MGB^c^ (417-429)	
ERBV1	5'-UTR of ERBV1 (NC_003983)	ERBV1 F	CCCC**TT**^f^CCCT**GA**AGATTGCT (148-167)	61
		ERBV1 R	GGCAAACGACCAACACATCA (190-209)	
		ERBV1 Pr	FAM-TTCTTCCAACTAAACCC-MGB (169-185)	
ERBV2	5'-UTR of ERBV2 (NC_003077)	ERBV2 F	CCCC**AA**CCCT**TG**AGATTGCT (148-167)	

^a^F = Forward primer. R = Reverse primer. Pr = Probe.

^b^Reporter dye (FAM; 6-carboxyfluorescein) labeled nucleotide.

^c^Nonfluorescent quencher dye (MGB™; minor groove binding) labeled nucleotide.

^d^K represents G or T.

^e^Y represents C or T.

^f^ Nucleotide difference between the forward primers of ERBV1 and ERBV2 are in bold.

The three assays were further evaluated for detection capability using 21 archived ERAV (n = 11) or ERBV (n = 10) isolates whose identity was previously confirmed in a one-way neutralization test using mono-specific rabbit antisera [16]. All three assays identified the ERV subtype accurately and no cross-reactivity between subtypes were observed. The mean Ct values of ERAV, ERBV1 and ERBV2 rRT-PCR assays are 24.53 ± 3.45 (ranging from 22 to 29), 29.50 ± 3.58 (ranging from 24 to 34) and 29.14 ± 5.22 (ranging from 18 to 35), respectively. Both ERBV1 and ERBV2 rRT-PCR assays could detect two previously well characterized ERBV1 isolates (NS CW and 58-13 NVS) [9]. None of the ERBV1 or ERBV2 rRT-PCR assays were able to distinguish viral RNA between ERBV1 and ERBV2 serotypes tested in the study. Overall, the two assays designed to distinguish ERBV1 and ERBV2 serotypes were able to detect the NVSL prototype strain of ERBV, indicating that the nucleotide mismatches in the forward primer were not sufficiently definitive to provide serotype specificity. This was further confirmed by their inability to distinguish the archived isolates as ERBV1 or ERBV2 serotype.

Previously Quinlivan *et al*. (2010) developed two TaqMan^®^ rRT-PCR assays targeting the conserved region of the 5'-UTR of the ERV genomes (Table 2) [33]. These two assays were also tested with RNA extracted from each of the two prototype strains of ERAV and ERBV, as well as TCF of 21 archived field isolates. The assay targeting ERAV could detect the ERAV prototype strain and the 11 ERAV positive isolates without cross-reacting with any ERBV samples. The mean Ct value was 15.31 ± 1.29 (ranging from 14 to 19), which was lower than the ERAV assay developed in our laboratory (24.53 ± 3.45, [ranging from 22 to 29]), indicating that this published ERAV assay is more sensitive in detecting the field isolates than the ERAV rRT-PCR assay developed in this study. In contrast, the assay targeting ERBV could not detect any of the ERBV isolates or the prototype strain of ERBV. This assay also did not cross-react with ERAV strains evaluated in this study. However, the reagents used in this study were not identical to those reported in the original publication by Quinlivan *et al*. (2010); this may also have contributed to the reduced sensitivity and failure to detect of ERBV RNA in the ERBV rRT-PCR assay. In order to provide a standardized protocol that can be easily applied in different laboratories, a commercial kit and manufacturer’s recommended real-time RT-PCR cycle parameters were used in the current study.

**Table 2:** **Primers and probes used in the rRT-PCR assay developed by Quinlivan*et al*. (2010)**

**rRT-PCR Assay Name**	**Target Genes (GenBank Accession Number)**	**Primer or Probe Names**^a^	**Sequence 5' to 3' and Nucleotide Location**	**Length of Fragment (bp)**
ERAV	5'-UTR of ERAV (NC_003982)	ERAV 468F	CCAGGTAACCGGACAGCG (468-485)	118
		ERAV 569R	GGCAGCGCTACCACAGG (569-585)	
		ERAV 508b	FAM^b^-CATTGCTCTGGATGGTGT-MGB^c^ (508-525)	
ERBV	5'-UTR of ERBV (NC_003983)	ERBV 77F	TGATGCTTGGCTCTCAGAAA (77-96)	132
		ERBV 189R	GCAAACGACCAACACATCAA (189-208)	
		ERBV 171b	FAM^b^-CTTCCAACTAAACCC-MGB^c^ (171-185)	

^a^F = Forward primer. R = Reverse primer. Pr = Probe.

^b^Reporter dye (FAM; 6-carboxyfluorescein) labeled nucleotide.

^c^Nonfluorescent quencher dye (MGB™; minor groove binding) labeled nucleotide.

### Development of cRT-PCR assays for the detection of ERAV and ERBV

**Figure 1:**
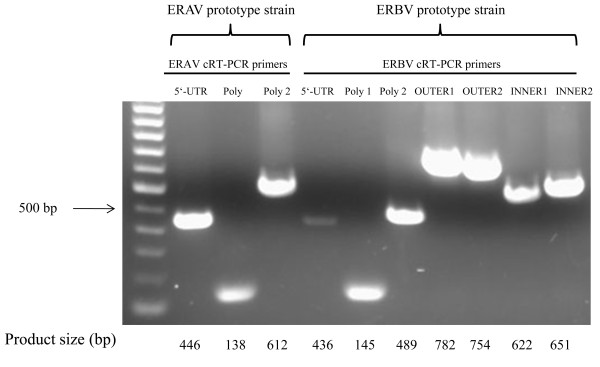
**Agarose gel electrophoresis of cRT-PCR products resulting from amplification RNA extracted from prototype strains of ERAV and ERBV from NVSL.** The individual RT-PCR product sizes are shown at the bottom of the figure.

To provide more diagnostic options, we developed an additional panel of six cRT-PCR assays to target the 5'-UTR and 3D^pol^ regions of ERAV and ERBV (Table 3). All primers were designed to distinguish ERAV and ERBV subtypes and were initially tested with the prototype strains of ERAV and ERBV obtained from NVSL. All primer pairs could detect the prototype strains for which they were designed and the cRT-PCR products matched their predicted sizes (Figure 1). The authenticity of the cRT-PCR products was confirmed by sequencing. Subsequently, the primers were tested with the RNA extracted from archived infective ERV TCF specimens. Similar to the rRT-PCR results, the ERAV 5'-UTR, ERAV Poly 1 and ERAV Poly 2 cRT-PCR assays could detect all the ERAV RNA from field isolates and did not cross-react with the RNA from ERBV isolates. The ERBV Poly 1 and ERBV Poly 2 cRT-PCR assays were also highly specific for the detection of RNA from ERBV isolates without cross-reacting with RNA from ERAV isolates. Sequence comparison analysis between the ERBV1 prototype P1436/71 (GenBank accession number X96871) and ERBV2 prototype P313/75 (GenBank accession number AF361253) revealed that these two strains shared 92.5% sequence identity in the 3D^pol^ region which is consistent with the previous findings that the 3D^pol^ region of ERBV is highly conserved and therefore commonly used for primer design [12,32]. The high sequence similarity between ERBV1 and ERBV2 in the 3D^pol^ region is good for primer design in differentiating ERBV from ERAV; however, it may also prevent the successful differentiation of ERBV1 and ERBV2 serotypes. In contrast to the high sensitivity of ERBV cRT-PCR assays targeting the 3D^pol^ region, the ERBV cRT-PCR assay targeting the 5'-UTR region could only detect 2 out of the 10 ERBV positive isolates (Table 4). All six assays were specific for ERV and did not react with other common equine respiratory viruses. Therefore, we concluded that the cRT-PCR assays that were developed could be used to distinguish ERAV from ERBV but not between ERBV serotypes.

**Table 3:** Primers used in the cRT-PCR assays

**cRT-PCR Assay Name**	**Primer Names**	**Sequence 5' to 3' and Nucleotide Location (nt)**	**Genbank Accession Number**	**Reference**
ERAV 5′UTR	ERAV 5′UTR F	TCAGCCCCCTGTCATTGACT (341-360)	NC_003982	This study
	ERAV 5′UTR R	TG**R**^a^TCAGGGCTGTAACCA (769-786)		
ERAV Poly	ERAV Poly F	TGGATGAAGTGGTTTTTGC (6384-6402)		
	ERAV Poly R	CAGTCAAAGCCTGGTTGTCA (6502-6521)		
ERAV Poly2	ERAV Poly F	TGGATGAAGTGGTTTTTGC (6384-6402)		
	ERAV Poly R2	ACTCTCATTGCATCAGCTGC (6977-6996)		
ERBV 5′ UTR	ERBV 5′UTR F	TTTCGTTCC**W**^b^CTTTAGC**R**^a^GG (349-368)	NC_003983	
	ERBV 5′UTR R	TCAGATCCGCACTCTATGAAG (764-784)		
ERBV OUTER1	ERBV OUTER F	TTTTGATGCTTCACATTCTCC (7986-8006)		[27]
	ERBV OUTER R	CGCTGTACCCTCGGTCCTACTC (8746-8767)		
ERBV OUTER2	ERBV OUTER F	TTTTGATGCTTCACATTCTCC (7986-8006)		
	ERBV INNER R	GCCTCGGCGAGTGAAGAG (8721-8739)		
ERBV INNER1	ERBV INNER F	CTTACTA**Y**^c^GAATGTGARGGGGC (8117-8138)		
	ERBV INNER R	GCCTCGGCGAGTGAAGAG (8721-8739)		
ERBV INNER2	ERBV INNER F	CTTACTAYGAATGTGARGGGGC (8117-8138)		
	ERBV OUTER R	CGCTGTACCCTCGGTCCTACTC (8746-8767)		
ERBV Poly1	ERBV Poly F	TTGAGTTGACCCTTCTGCA (7409-7427)		This study
	ERBV Poly R	TCATACTCTGAAATG**R**^**a**^**K**^**d**^TCCATTG (7530-7553)		
ERBV Poly2	ERBV Poly F	TTGAGTTGACCCTTCTGCA (7409-7427)		
	ERBV Poly R2	GCTGAACCAATGCCTAATCC (7879-7898)		

^a^ R represents A or G.

^b^ W represents A or T.

^c^ Y represents C or T.

^d^ K represents G or T.

**Table 4:** Archived ERAV and ERBV isolates tested with rRT-PCR assays and cRT-PCR assays

**Sample**	**Source of TCF**	**rRT-PCR Assays**			**cRT-PCR Assays**									
		**ERAV**	**ERBV**		**ERAV**			**ERBV**						
		**ERAV**	**ERBV1**	**ERBV2**	**ERAV 5'-UTR**	**ERAV Poly**	**ERAV Poly 2**	**ERBV 5'-UTR**	**ERBV Poly 1**	**ERBV Poly 2**	**ERBV OUTER 1**^c^	**ERBV OUTER 2**^c^	**ERBV INNER 1**^c^	**ERBV INNER 2**^c^
**Serotype ERAV**														
ERAV	NVSL^a^	+	−	−	+	+	+	−	−	−	−	−	−	−
PERV, P4 2004 A	GERC^b^	+	−	−	+	+	+	−	−	−	−	−	−	−
Plowright,P4 2004 A	GERC	+	−	−	+	+	+	−	−	−	−	−	−	−
T3 isolate P10/2004 A	GERC	+	−	−	+	+	+	−	−	−	−	−	−	−
T10 isolate P9/2004 A	GERC	+	−	−	+	+	+	−	−	−	−	−	−	−
945 isolate P4/2004 A	GERC	+	−	−	+	+	+	−	−	−	−	−	−	−
ERV-1 (A) Plowright P4	GERC	+	−	−	+	+	+	−	−	−	−	−	−	−
ERV-1 (A) PERV P4	GERC	+	−	−	+	+	+	−	−	−	−	−	−	−
Amp 87-73-69-945	GERC	+	−	−	+	+	+	−	−	−	−	−	−	−
NS-T3	GERC	+	−	−	+	+	+	−	−	−	−	−	−	−
NS-T10	GERC	+	−	−	+	+	+	−	−	−	−	−	−	−
U-187	GERC	+	−	−	+	+	+	−	−	−	−	−	−	−
**Serotype ERBV**														
ERBV	NVSL	−	+	+	−	−	−	+	+	+	+	+	+	+
Swiss isolate P6 2004 B	GERC	−	+	+	−	−	−	−	+	+	+	+	+	+
NS CW	GERC^d^	−	+	+	−	−	−	+	+	+	+	+	+	+
U-198V	GERC	−	+	+	−	−	−	−	+	+	+	+	+	+
NS-SD	GERC	−	+	+	−	−	−	−	+	+	+	+	+	+
Mare 189	GERC	−	+	+	−	−	−	−	+	+	+	+	+	+
51-12NVS	GERC	−	+	+	−	−	−	−	+	+	+	+	+	+
57-10NVS	GERC	−	+	+	−	−	−	−	+	+	+	+	+	+
57-11NVS	GERC	−	+	+	−	−	−	−	+	+	+	+	+	+
57-13NVS	GERC	−	+	+	−	−	−	−	+	+	+	+	+	+
58-13 NVS	GERC^d^	−	+	+	−	−	−	−	+	+	+	+	+	+

^a^ National Veterinary Services Laboratories, Ames, IA.

^b^ Maxwell H. Gluck Equine Research Center. All archived samples from GERC were obtained from the late Dr. William H. McCollum.

^c^ These assays were developed by Black *et al*. (2007).

^d^ These two TCF samples were also included in a study done by Black *et al*. (2005).

Previously Black *et al*. (2007) developed four primers for the detection of ERBV RNA by RT nested-PCR assay [27]. We took these four primers and mix and matched them depending on their positions to generate four one-step cRT-PCR assays (Table 3). These four assays (ERBV OUTER 1, ERBV OUTER 2, ERBV INNER 1 and ERBV INNER 2) could distinguish all ERBV positive isolates from ERAV isolates included in this study. The application of one-step RT-PCR has a greater advantage over the nested RT-PCR because it eliminates the possibility of cross-contamination between samples and reduces the turnaround time.

### Comparison of sensitivities of the rRT-PCR and cRT-PCR assays

**Figure 2:**
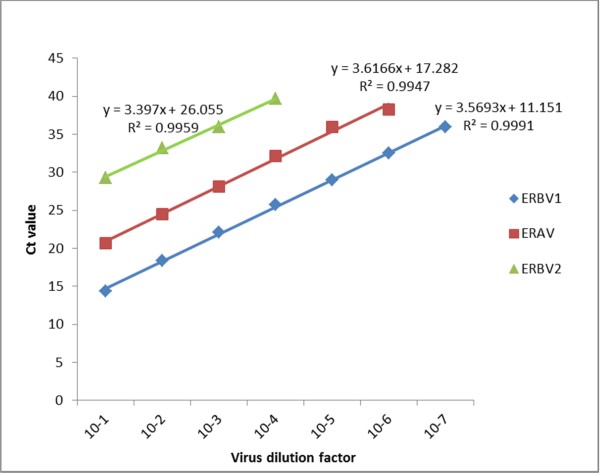
**Comparison of detection sensitivity of the three rRT-PCR assays using ERAV or ERBV prototype strains from NVSL (ERAV rRT-PCR assay [*y*** **= 3.4543*x*** **+ 17.373,*R***^***2***^ **= 0.9949], ERBV1 rRT-PCR assay [*y*** **= 3.4682*x*** **+ 11.719,*R***^***2***^ **= 0.9997] and ERBV2 rRT-PCR assay [*y*** **= 3.397*x*** **+ 26.055,*R***^***2***^ **= 0.9959]).**

Using serial decimal dilutions (10^−1^ to 10^−10^) of the TCF containing ERAV and ERBV prototype strains, the detection limits of all rRT-PCR and cRT-PCR assays were compared (Table 5, Figure 2). Viral RNA from each of the serial dilutions was eluted in 50 μl of nuclease free water and 5 μl was tested in duplicate in both rRT-PCR and cRT-PCR assays. The plaque number in the highest dilution was used to calculate the number of infectious particles that can be detected by each assay. The amplification efficiency of the three ERAV, ERBV1 and ERBV2 rRT-PCR assays was 97.0%, 94.8% and 94.2%, respectively, calculated according to a previously described method (Figure 2) [34]. The ERAV rRT-PCR assay and ERAV 5'-UTR cRT-PCR assay could detect viral RNA up to 10^−6^ dilution of ERAV in TCF which is approximately 1 pfu/ml infectious virus particles. The other two ERAV cRT-PCR assays (ERAV Poly1 and ERAV Poly 2) were 10-fold less sensitive (10^−5^ virus dilution, which is approximately 10 pfu/ml) as compared to the rRT-PCR and cRT-PCR assays targeting the 5'-UTR regions (Figure 2). The ERBV1 rRT-PCR assay could detect ERBV viral RNA up to 10^−7^ dilution which equals 1.2 pfu/ml infectious virus particles. The third rRT-PCR assay targeting ERBV2 was 3 logs less sensitive compared to the ERBV1 rRT-PCR assay and could only detect more than 550 pfu/ml infectious virus particles. This suggests that the ERBV1 rRT-PCR assay is more suitable for the detection of ERBV in clinical samples. All the ERBV cRT-PCR assays except ERBV 5'-UTR cRT-PCR assay could detect 10 to 30 pfu/ml infectious virus particles (10^−5^ to 10^−6^ dilutions). The ERBV 5'-UTR cRT-PCR assay was the least sensitive and could only detect virus in the 10^−1^ dilution of TCF (2.75 × 10^5^ pfu/ml infectious virus particles).

**Table 5:** **Detection limit of virus particles with rRT-PCR assays and cRT-PCR assays**^**a**^

**ERV Strains**^b^	**TCF Titer**	**Assay name**												
		**rRT-PCR Assays**			**cRT-PCR Assays**									
		**ERAV**	**ERBV**		**ERAV**			**ERBV**						
		**ERAV**	**ERBV1**	**ERBV2**	**ERAV 5'-UTR**	**ERAV POLY1**	**ERAV POLY2**	**ERBV 5'-UTR**	**ERBV OUTER 1**^d^	**ERBV OUTER 2**^d^	**ERBV INNER 1**^d^	**ERBV INNER 2**^d^	**ERBV Poly 1**	**ERBV Poly 2**
ERAV	10^−7^	10^−6^	NA^c^	NA	10^−6^	10^−5^	10^−5^	NA	NA	NA	NA	NA	NA	NA
ERBV	10^−7^	NA	10^−7^	10^−4^	NA	NA	NA	10^−1^	10^−5^	10^−4^	10^−4^	10^−4^	10^−4^	10^−5^

^a^ Serial decimal dilutions of ERAV and ERBV were tested in a comparison study by virus isolation in cell culture, rRT-PCR and standard RT-PCR assays. Numbers shown on the table represent the serial virus dilution.

^b^ ERAV and ERBV prototype strains were obtained from NVSL.

^c^ Not applicable.

^d^ The primers used in these four assays were obtained from a nested RT-PCR developed by Black *et al*. (2007).

Overall, the rRT-PCR assays were more sensitive than the cRT-PCR assays. There was approximately a 10-fold difference in the limit between the rRT-PCR and cRT-PCR assays in detecting ERAV strains. There was a significant difference in the sensitivity of the rRT-PCR and cRT-PCR assays targeting the ERBV strains. The ERBV 5'-UTR cRT-PCR assay was the least sensitive among all the developed assays. This might be explained by the fact that the reverse primer of the ERBV 5'-UTR cRT-PCR assay was located in the higher order internal ribosome entry site [7]. This complex sequence region may prevent efficient binding of the reverse primer to target sequences. However, no plausible explanation can be provided for the high sensitivity of the ERAV 5'-UTR cRT-PCR assay which was targeting the similar region as compared to ERBV.

Although ERAV and ERBV infections are considered a common disease in horses, limited data are available about the pathogenesis and disease prevalence, which may be due in part to the absence of suitable diagnostic methods for these infections [13-19,28,35,36]. Currently, there are only a few molecular diagnostic tests available for the detection of these viruses including a duplex rRT-PCR developed by Mori *et al*. (2009) [32] and single rRT-PCR assays for ERAV or ERBV developed by Quinlivan *et al*. (2010) [33]. The duplex rRT-PCR by Mori *et al*. (2009) was developed to differentiate ERAV from ERBV, but when tested, none of the samples were positive for ERAV [32]. Therefore the detection capability for ERAV using this duplex rRT-PCR assay is questionable. The single rRT-PCR assays developed by Quinlivan *et al*. (2010) detected 30 ERAV and 5 ERBV positives among 300 nasal swab samples collected over a 7 year period [33]. As we discussed above, the ERBV rRT-PCR assay developed in that study was unable to detect any ERBV isolates tested in this study using the current cycling conditions and rRT-PCR reagents. The assays developed in our study were tested with a limited number of well-characterized ERAV and ERBV isolates [9,27]. Therefore, the authors of this manuscript admit that prior to the application of the assays on a routine diagnostic basis, both would need to be more fully evaluated using a larger number of clinical specimens positive for both ERAV and ERBV.

## Conclusions

In the current study, we developed one rRT-PCR assay and three cRT-PCR assays for the detection of ERAV and three rRT-PCR assays and three cRT-PCR assays for ERBV. Twenty-one archived ERAV or ERBV field isolates were used to evaluate the detection capability of the assays. Both the rRT-PCR and cRT-PCR assays designed for ERAV or ERBV could detect the serotype specific isolates without cross-reacting with other equine viral pathogens. Comparison of the respective sensitivities of rRT-PCR assays and cRT-PCR assays confirmed that the rRT-PCR assays have the same or greater sensitivity in detecting ERV in serial decimal dilutions of infective TCFs. Overall, the newly developed assays provide valuable tools for the detection of ERAV and ERBV.

## Methods

### Cells and viruses

The high passage RK-13 cell line (RK-13 KY; passage level 399-409) was maintained in Eagle’s minimum essential medium (EMEM) supplemented with 10% ferritin-supplemented bovine calf serum (Hyclone Laboratories, Inc., Logan, UT), 1% penicillin and streptomycin, and 0.1% amphotericin B (1,000 μg/ml). The overlay medium used for inoculated cultures was 0.75% carboxymethylcellulose (CMC) (Sigma-Aldrich, St. Louis, MO) in supplemented EMEM. All of the other reagents were obtained from Mediatech, Inc., Herndon, VA.

Twenty-one TCF samples containing ERAV, ERBV1 and ERBV2 were included in this study. These samples were previously isolated from nasal swabs and urine samples from horses and characterized by the late Dr. William H. McCollum at the Maxwell H. Gluck Equine Research Center, University of Kentucky. The prototype strains of ERAV (NVSL-0600EDV8501) and ERBV (NVSL-0610EDV85010) from NVSL, Ames, IA were also included in the study. Virus working stocks were produced by propagating the viruses in RK-13 cells as described previously.

To determine the specificity of the rRT-PCR and cRT-PCR assays, viral nucleic acid from each of the following equine viral pathogens was included in the study: equine arteritis virus (ATCC VR-796), equine herpesviruses 1-5 (EHV-1 [ATCC VR-700], EHV-2 [ATCC VR-701], EHV-3 [ATCC VR-352], EHV-4 [ATCC VR-2230], and EHV-5 [37]), equine adenovirus 1 (NVSL-001EDV8401) and 2 (University of Kentucky Veterinary Diagnostic Laboratory), equine influenza virus (EIV) type A1 (equine/Prague/1/56 [H7N7]; NVSL-021IDV9201) and A2 (equine/Miami/63/[H3N8]; NVSL-060IDV0501], equine/Kentucky/81 [H3N8; NVSL-040IDV0001], equine/Alaska//91 [H3N8; NVSL-020IDV9101]), and Salem virus, a novel paramyxovirus of horses [38]. The EHV-5 and Salem virus were kindly provided by Dr. Stephen Bell at University of California, Davis, CA and Dr. Edward Dubovi, Cornell University, Ithaca, NY, respectively.

### RNA extraction

Viral RNA was prepared from virus-infective tissue culture fluid (TCF) using the MagMAX™-96 Viral RNA Isolation Kit (Applied Biosystems, Forest City, CA) according to the manufacturer’s instructions. Briefly, TCF samples were microcentrifuged at 13,800 × g for 2 min, and 50 μl of supernatant was removed and used for nucleic acid extraction. The viral nucleic acid was eluted in 50 μl nuclease free water and stored at −80°C.

### Primers and probes

The conserved and variable regions of each equine rhinitis virus serotype (ERAV and ERBV) have been determined by alignment of 12 sequences (8 ERAV [GenBank accession numbers: L43052, DQ272127, NC_003982, X96870, DQ272578, DQ272577, DQ268580 and DQ272128], 2 ERBV1 [GenBank accession numbers: NC_003983 and X96871] and 2 ERBV2 [GenBank accession numbers: AF361253 and NC_003077]) available in GenBank. The rRT-PCR fluorescent TaqMan^®^ MGB™ (minor groove binding) probes and forward and reverse primers for ERAV, ERBV1 and ERBV2 were designed to target the conserved regions in 5'-UTR of each strain using Primer Express™ software (Applied Biosystems, Foster City, CA) (Table 1). Similarly, the cRT-PCR forward and reverse primers from both serotypes were designed to target the 5'-UTR and 3D^pol^ regions of the genome using Vector NTI (Applied Biosystems, Foster City, CA). The four primers from a nested RT-PCR assay developed by Black *et al*. (2007) were included in the study as four, one-step cRT-PCR assays. Two rRT-PCR assays described by Quinlivan *et al*. (2010) were compared to the assays described in this manuscript (Table 2).

### One-step rRT-PCR assay

The one-step TaqMan^®^ rRT-PCR assay was performed using the TaqMan One-Step RT-PCR Master Mix in a 7500 Fast Real-Time PCR System as previously described [39]. Every sample was tested in duplicate in each assay. Briefly, 25 μl of RT-PCR mixture for each reaction contained 12.5 μl of 2 × Master Mix without UNG (uracil-N-glycosylase), 0.625 μl of 40 × MultiScribe and RNase Inhibitor Mix, 0.45 μl of 50 μM forward and reverse primers (final concentration 900 nM), 0.625 μl of 10 μM probe (final concentration 250 nM), 5.35 μl of nuclease free water, and 5 μl of test sample RNA. The following thermocycling conditions were used under standard mode as per manufacturer’s recommendation: 30 min at 48°C, 10 min at 95°C, followed by 40 cycles at 95°C for 15 sec and 60°C for 1 min. Each RT-PCR run included a control without RNA (containing the reaction mix with 5 μl of water [no template control]) and positive controls containing ERAV or ERBV RNA.

### cRT-PCR assay and sequencing analysis

The cRT-PCR was performed using a Qiagen OneStep RT-PCR kit (Qiagen, Santa Clara, CA) and 5 μl of test sample RNA in a mastercycler gradient thermal cycler (Eppendorf, Westbury, NY) according to the manufacturer’s recommendation. Briefly, 50 μl of RT-PCR reaction contains 10 μl of 5 × Qiagen OneStep RT-PCR buffer, 2 μl of Qiagen OneStep RT-PCR enzyme mix, 2 μl of 10 mM dNTP mix (final concentration 0.4 mM), 1 μl of RNase Inhibitor, 1 μl of each of the forward and reverse primers, 28 μl of nuclease free water and 5 μl of RNA template. The following thermal cycler conditions were used: 50°C for 30 min, 95°C for 15 min, followed by 40 cycles of 30 sec 94°C denaturation, 30 sec 50°C annealing, 1 min 72°C extension with a 10 min 72°C final extension.

The authenticity of the cRT-PCR products amplified from ERAV or ERBV NVSL prototype strains were sequenced using the primers which were used to amplify the products. The sequence data were analyzed using Aligner version 1.5.2 (CodonCode, Dedham, MA) software program.

### Determination of detection limits of rRT-PCR and cRT-PCR assays

Using serial decimal dilutions (10^−1^ to 10^−10^) of ERAV and ERBV prototype strains (NVSL-0600EDV8501 and NVSL-0610EDV85010, respectively), the detection limits of the rRT-PCR and cRT-PCR assays were evaluated. To minimize inter-assay variability, equal aliquots of each dilution were used in all three assays. Briefly, 5 μl of RNA extracted from 50 μl of each decimal dilution were used in rRT-PCR and cRT-PCR assays as described above. RNA was run in duplicate in rRT-PCR assays, and both cRT-PCR and rRT-PCR assays were repeated three times independently.

## Authors’ contributions

UBR designed the study and supervised all the laboratory procedures. ZL extracted nucleic acids, developed and performed both rRT-PCR and cRT-PCR assays with prototype viruses. She wrote the manuscript with UBR. JW along with ZL performed rRT-PCR and cRT-PCR assays using archived TCF samples. PJT along with the late Dr. McCollum performed the isolation and characterization of ERAV and ERBV used in this study. All authors read and approved the final manuscript.
